# Strange Admonition of Death

**Published:** 1887-05

**Authors:** 


### STRANGE ADMONITION OF DEATH.

A few days ago, says *The Salt Lake Herald*, we chronicled the death of Miss Athaliah Gilbert, of South Cottonwood. At the time of the announcement there were reports current that some events out of the ordinary were connected with her disease, but at that time there were no means to ascertain the particulars. Yesterday, however, Mr. James Gilbert, the young lady's father, and several other Cottonwood people were in the city, and from them a reporter learned the facts which follow. All the names mentioned are those of responsible and well-known citizens, and unreal as the narration sounds, there can be no doubt of its authenticity.

The young lady was sixteen years old at the time of her death, and appears to have been possessed of one of those warm, lovable, bright, and even-tempered dispositions, which endear the owner to everyone with whom she comes in contact. Though so young, she took a busy part in all church duties, and in improvement associations and the Sunday-school her name always had a prominent place. Some three or four years ago she formed an intimacy with a youth named John Cunliffe, the son of a neighbor, and despite the tender years or both, they became strongly attached to each other, and provoked no end of comment at their old-fashioned devotion and steadfast affection for one another. This state of affairs continued until she was fifteen years old, when the association was rudely broken by the death of young Cunliffe. He lost his life from the kick of a wild horse about a year ago. When the intelligence was brought to Miss Gilbert, her father says, it gave her a shock from which she never recovered. She almost sank beneath the blow, and at his funeral her paroxysms of grief were so violent that it was feared her reason would depart. In time, however, she resumed her accustomed duties, but it was evident that the blow she had sustained had sunk deep into her life. She seldom roused herself from a deep lethargy of sadness and day by day her color and strength and the freshness of youth seemed to be ebbing away. A few months ago she alarmed her sister by telling her that "John" had visited her chamber and had told her that she must prepare to come to him. She manifested no fear, but, according to her sister, had told him she could not leave her parents, but he had only said that she must come. Once again, later, she told her sister that he had come to her with the same message, and she had now, evidently, given up desiring to remain, as she told her sister how she wished to be dressed at her burial, and whom she wished to dress her. Soon after that, young Cuneliffe's father came to Mr. Gilbert, sorely disturbed, and told him that one morning as he was lying down his son had come to him and stood at the foot of his bed. His father had asked him what it was that he desired, and he replied: "I came to see you, father. I am staying at Gilbert's and I am going back there now. I have been there ever since I left you. Where else should I be?" Mr. Gilbert attempted to reason the old gentleman out of his notion, but he insisted that it was no dream or vision, but that his son had actually visited and spoken to him, and that in broad daylight. In the meantime, Miss Gilbert continued to maintain that her last day was approaching, and no amount of persuasion seemed to shake her belief. One week ago last evening she and her parents were attending a birthday party at a neighbor's. Miss Gilbert was sitting at the lunch table, chatting with some companions, when, without a word of warning, she fell to the floor motionless. Her father and mother raised her, and both said her heart had ceased to beat. Their cries and lamentations and their frenzied attempts to rouse her, they state, rallied her for a few moments, and she was hurriedly conveyed home, where she expired shortly afterward, leaving her friends almost stupefied with grief. Her funeral was one of the largest convocations of mourners ever seen in that locality.

